# What a model: A newly developed pancreatic murine cell line permissive to adenoviral replication

**DOI:** 10.1016/j.omton.2025.200956

**Published:** 2025-03-13

**Authors:** Manlio Fusciello, Vincenzo Cerullo

**Affiliations:** 1 Drug Research Program (DRP), ImmunoViroTherapy Lab (IVT), Division of Pharmaceutical Biosciences, Faculty of Pharmacy, University of Helsinki, Viikinkaari 5E, 00790 Helsinki, Finland; 2 Helsinki Institute of Life Science (HiLIFE), University of Helsinki, Fabianinkatu 33, 00710 Helsinki, Finland; 3 Translational Immunology Program (TRIMM), Faculty of Medicine Helsinki University, University of Helsinki, Haartmaninkatu 8, 00290 Helsinki, Finland; 4 Digital Precision Cancer Medicine Flagship (iCAN), University of Helsinki, 00014 Helsinki, Finland; 5 Department of Molecular Medicine and Medical Biotechnology and CEINGE, Naples University Federico II, 80131 Naples, Italy

## Main text

The pursuit of effective therapies for pancreatic ductal adenocarcinoma (PDAC) remains a formidable challenge in oncology because of poor prognosis and resistance to conventional treatments. As a result, the field has turned to innovative approaches, such as oncolytic adenoviruses (OAs). One type of OA, conditionally replicating adenoviruses (CRAds), can replicate within tumor cells and induce tumor cell lysis but leave healthy cells intact. The selectivity of CRAds is achieved by deleting adenoviral genes encoding cell cycle regulatory proteins and/or inserting a tissue-specific promoter to control an essential viral replication gene.^1^^,^^2^ CRAds have shown potential in preclinical and clinical studies, offering dual benefits of direct tumor lysis and immune stimulation. However, the lack of suitable immunocompetent murine models that are permissive to human adenovirus replication has remained a major barrier.

Most preclinical evaluations of OAs rely upon xenograft models in immunodeficient mice or partially permissive animal models such as Syrian hamsters.^3^^,^^4^ For example, human adenovirus serotype 5 (Ad5) can replicate in several hamster cell lines, but the burst size is 7-fold less than that observed in the human A549 lung cancer cell line (one of the most permissive cell lines available).^3^ Moreover, a lack of widely available genetic tools and immune profiling makes it difficult to investigate the interplay between virotherapy and host immune responses. These limitations have mostly restricted assessments of OA efficacy to less clinically relevant immunodeficient mouse models. However, in this issue of *Molecular Therapy Oncology*, Otero-Mateo et al. introduce a new pancreatic cancer cell line, KPC-I, that can support human adenoviral replication in immunocompetent mice, thus allowing investigation of the intricate balance between antitumor and antiviral immune responses.

Similar to the semi-permissive KLN205 and CMT64 cell lines, the KPC-1 cell line is permissive to adenoviral replication, which allows for a thorough investigation of OAs in a clinically relevant tumor environment.^5^^,^^6^^,^^7^^,^^8^ Adenoviral infection of KPC-1 cells demonstrates robust antitumor effects in both immunocompetent and immunodeficient settings. Moreover, tumor regression occurs following the administration of two different adenoviral vectors, AdNuPARE1A and ICOVIR15kDelE3, demonstrating viral replication, immune activation, and therapeutic efficacy in KPC-1 xenografts.

These findings demonstrate the successful application of OAs to an improved preclinical model of pancreatic cancer. By leveraging both the antiviral response and the broader antitumor activity of the immune system, this approach offers a promising avenue for enhancing therapeutic efficacy. Given the urgent need for more effective treatment options in pancreatic cancer, this strategy could create significant opportunities for improved clinical outcomes. Otero-Mateo et al. overcome a long-standing species barrier, opening new avenues for studying OA-driven tumor immunology and developing next-generation virotherapies. Future studies leveraging the KPC-I model will undoubtedly refine our understanding of viral-host interactions and may improve therapeutic outcomes in pancreatic cancer in the future.

## Author contributions

The authors have contributed equally to the manuscript.

## Declaration of interests

The authors have no conflicts of interest.
